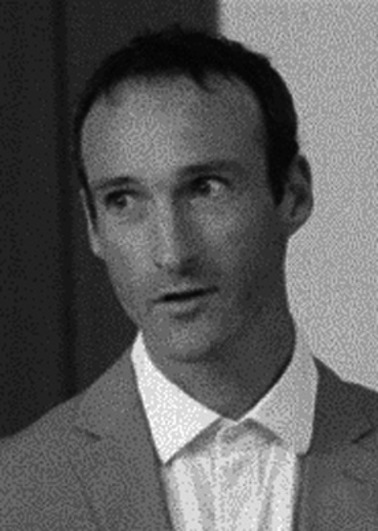# What is Next in Anion‐Exchange Membrane Water Electrolyzers? Bottlenecks, Benefits, and Future

**DOI:** 10.1002/cssc.202200027

**Published:** 2022-03-24

**Authors:** Carlo Santoro, Alessandro Lavacchi, Piercarlo Mustarelli, Vito Di Noto, Lior Elbaz, Dario R. Dekel, Frédéric Jaouen

**Affiliations:** ^1^ Department of Materials Science University of Milano-Bicocca U5, Via Cozzi 5 20125 Milano Italy; ^2^ Istituto di Chimica Dei Composti OrganoMetallici (ICCOM) Consiglio Nazionale Delle Ricerche (CNR) Via Madonna Del Piano 10 50019 Sesto Fiorentino Firenze Italy; ^3^ Section of Chemistry for the Technology (ChemTech) Department of Industrial Engineering University of Padova Via Marzolo 9 I-35131 Padova PD Italy; ^4^ Department of Chemistry and the Institute of Nanotechnology and Advanced Materials Bar-Ilan University Ramat-Gan 5290002 Israel; ^5^ The Wolfson Department of Chemical Engineering Technion – Israel Institute of Technology Haifa 3200003 Israel; ^6^ The Nancy & Stephen Grand Technion Energy Program (GTEP) Technion – Israel Institute of Technology Haifa 3200003 Israel; ^7^ ICGM Univ. Montpellier CNRS, ENSCM Montpellier France

**Keywords:** anion-exchange membrane, electrocatalysis, electrolyzers, platinum-group metal-free, water electrolysis

## Abstract

As highlighted by the recent roadmaps from the European Union and the United States, water electrolysis is the most valuable high‐intensity technology for producing green hydrogen. Currently, two commercial low‐temperature water electrolyzer technologies exist: alkaline water electrolyzer (A‐WE) and proton‐exchange membrane water electrolyzer (PEM‐WE). However, both have major drawbacks. A‐WE shows low productivity and efficiency, while PEM‐WE uses a significant amount of critical raw materials. Lately, the use of anion‐exchange membrane water electrolyzers (AEM‐WE) has been proposed to overcome the limitations of the current commercial systems. AEM‐WE could become the cornerstone to achieve an intense, safe, and resilient green hydrogen production to fulfill the hydrogen targets to achieve the 2050 decarbonization goals. Here, the status of AEM‐WE development is discussed, with a focus on the most critical aspects for research and highlighting the potential routes for overcoming the remaining issues. The Review closes with the future perspective on the AEM‐WE research indicating the targets to be achieved.

## Graphical abstract

**Water electrolysis**: In this Review, the advantages and challenges of anion‐exchange membrane water electrolyzers are discussed. The status of their development with a focus on the most critical aspects for research is presented, highlighting the potential routes for overcoming the remaining issues, and future targets to be achieved are also presented.

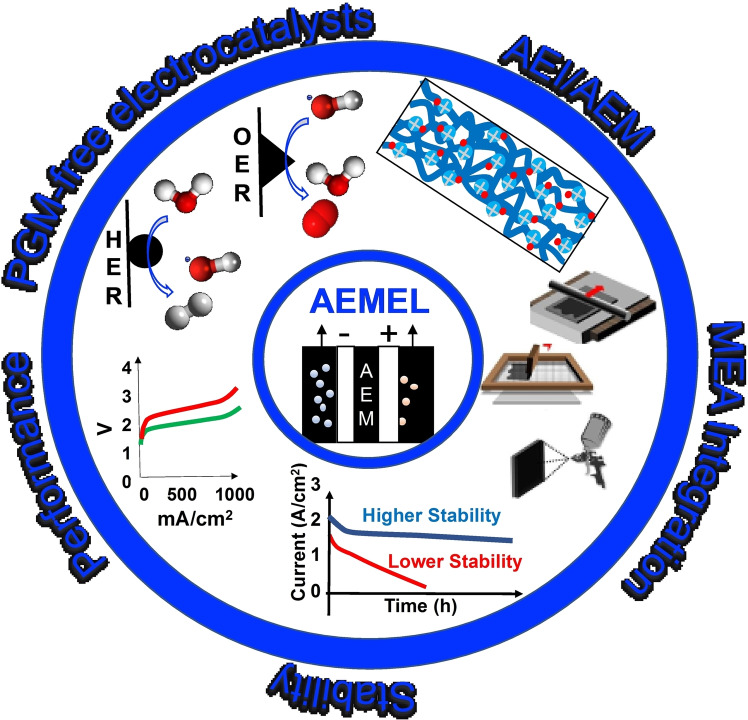

## 1 Introduction

Global climate change is calling for a paradigm shift in the way energy is produced, stored, and used. Hydrogen as an energy vector with low environmental impact will certainly play an important role in the global decarbonization goals set for 2050.^[1]^ Several routes and technologies for producing green hydrogen exist.^[2]^ However, the only route capable, in principle, of satisfying the massive industrial and private demands is water electrolysis, powered by green electricity produced by sustainable energy sources.[^2^, ^3^]

The Hydrogen Economy substantially relies on electrochemical water electrolysis. Low‐, intermediate‐, and high‐temperature water electrolyzers are commercially available. However, intermediate‐ and high‐temperature electrolyzers operate best with constant load and cannot be turned on/off easily. This, in turn, is a drawback for their coupling with wind/solar electricity, which will account for a large share of renewable electricity in the future.[^4^, ^5^]

Two low‐temperature water electrolysis technologies are currently available on the market: alkaline water electrolyzers and proton‐exchange membrane water electrolyzers.^[5]^ Since there is no unique or standard acronym to describe these two technologies, in this Review the acronyms of A‐WE and PEM‐WE are used to specifically refer to alkaline water electrolyzers and proton exchange membrane water electrolyzers, respectively.

A‐WEs work in KOH‐rich electrolyte and can operate without platinum‐group metal (PGM) electrocatalysts. However, A‐WEs suffer from problems related to safety, operation, and low efficiency, incapable of producing pressurized hydrogen, in turn needed for automotive applications. In contrast, PEM‐WEs are efficient but rely on electrocatalysts based on scarce elements such as iridium [to catalyze the anodic oxygen evolution reaction (OER)], which is one of the rarest metal on Earth, and platinum [to catalyze the cathodic hydrogen evolution reaction (HER)]. In 2011, the EU identified both these elements as critical raw materials (CRMs) due to their scarcity, cost, and risk of supply.^[6]^

Considering a broad deployment of PEM‐WE devices for mass production of green hydrogen worldwide, the current supply chain of CRMs will not satisfy the growing market demand, and as a result, the price of these elements (which are already extremely expensive compared to, e. g., any metal from the first row of transition metals) would skyrocket and impede the production and implementation at the required scale.^[7]^ Focusing water electrolysis technologies on PGM electrocatalysts that are: (i) rare elements, (ii) geographically located mainly in two countries (South Africa, Russia), (iii) with unpredictable cost fluctuation, is risky and, in some cases, unethical.

In addition, PEM‐WEs currently lean on fluorine‐based polymers, with strong environmental impact because of the emission of fluorocarbon gases at the production stage of tetrafluoroethylene. The phasing‐out of fluoropolymers for environmental reasons has been recently proposed, and the European Commission is currently active in restricting their use in the near future.[^8^, ^9^]

A water electrolysis technology that might combine the advantages of A‐WE and PEM‐WE is the anion‐exchange membrane water electrolyzer (AEM‐WE). By our definitions, the key difference between A‐WE and AEM‐WE is that the electrode separator in the former technology has no intrinsic ionic conductivity, its conductivity being provided by KOH filling the pores of a porous separator in A‐WE, while in AEM‐WE, the polymeric membrane is non‐porous and possesses intrinsic anionic conductivity. Yet, most studies on AEM‐WE are currently resorting to dilute KOH electrolyte support for reaching high performance, as discussed in detail later.

AEM‐WE is already more efficient compared to A‐WE and has the potential to operate in high‐pH environments without CRM and PGM, and without alkaline electrolyte.[^10^, ^11^, ^12^, ^13^, ^14^] AEM‐WEs have key advantages over A‐WEs: (i) they operate at higher current densities due to lower ohmic resistance and consequently have a smaller volumetric footprint while utilizing fewer materials that in turn lead to a smaller carbon footprint; (ii) they allow pressurizing hydrogen electrochemically, thus facilitating its subsequent storage, distribution, and final utilization; (iii) with a non‐porous polymeric membrane, the safety of the system is significantly improved. Moreover, AEM‐WEs have key advantages over PEM‐WEs of: (i) reducing the EU dependence on CRMs throughout the entire balance of materials, and (ii) reducing the environmental footprint over the entire life cycle of the product.

However, the AEM‐WE technology is a relatively new technology and faces many issues that have to be solved before fulfilling its full potential. It not only needs to close the existing gap with PEM‐WE in terms of efficiency and durability, but it must show that it is possible to do so with CRM‐free electrocatalysts (especially on the anode side) and cell components (mainly, bipolar plate and porous media).

Several Reviews on the topic of AEM‐WEs were recently published and detailed below. The development of PGM‐ and/or CRM‐free electrocatalysts for the HER and OER in alkaline medium was described recently.[^10^, ^11^, ^12^, ^13^, ^14^, ^15^, ^16^, ^17^] The recent development of AEM and anion‐exchange ionomers for AEM‐WE or AEM fuel cell application was reviewed in Refs. [18–22]. Particularly, AEMs developed for AEM‐WE application were summarized in Refs. [18, 19]. Lim et al. recently presented a Review related to radiation‐grafted AEMs for fuel cells and electrolyzers.^[20]^ In Ref. [21], a comprehensive Review related to the structures and properties of polymers in anion and cation exchange membranes for water electrolysis application is presented. Advances in AEMs for fuel cells and electrolyzers application were also presented by Mandal.^[22]^

While many Reviews can be found on the development of separate AEM‐WE components, the investigation of such novel functional materials in AEM‐WE device is more recent, and consequently, only few Review articles on the performance of such materials in AEM‐WE have been published hitherto. In Ref. [23], the performance and durability of AEM‐WE with PGM‐free OER anodes was presented. A recent Review has also highlighted the durability‐limiting factors that restrict the long‐term use of AEM‐WEs.^[24]^ Miller et al. recently published a Review article on green hydrogen produced from AEM‐WE, with particular focus on the recent developments in crucial materials and operating conditions.^[25]^ Comprehensive overviews of AEM‐WE technologies were also recently presented,^[26]^ and a roadmap was released in 2019.^[27]^ A Review of commercially available AEMs and their performance in AEM‐WE was reported by Henkensmeyer et al.^[28]^ A short Review including a survey of the development of separate components (HER and OER electrocatalysts, membranes) and their integration in membrane electrode assembly (MEA) and testing in AEM‐WE was recently presented.^[29]^

In this Review, we (i) shortly describe A‐WEs and PEM‐WEs identifying the state of the art and their limitation; (ii) highlight the differences between A‐WEs, PEM‐Wes, and AEM‐WEs identifying advantages and disadvantages; (iii) describe the state‐of‐the‐art AEMs, indicating the drawbacks and the possible solutions; (iv) describe HER and OER electrocatalysts state‐of‐the‐art performance, indicating the bottlenecks and the possible solutions; (v) describe the porous transport layer (PTL) reporting the related problems and its crucial role within AEM‐WEs; (vi) propose activities to overcome limitations identifying the challenges, related benefits, and achievements; (vii) present an outlook on research needs and opportunities. This Review aims to identify the drawbacks and discuss how these limitations may be overcome, indicating the roadmap towards the successful large‐scale deployment of AEM‐WE technology. Finally, quantified targets for the entire AEM‐WE are identified and described.

## 2 Water Electrolysis Technologies

### 2.1 Short description

**Figure 1:**
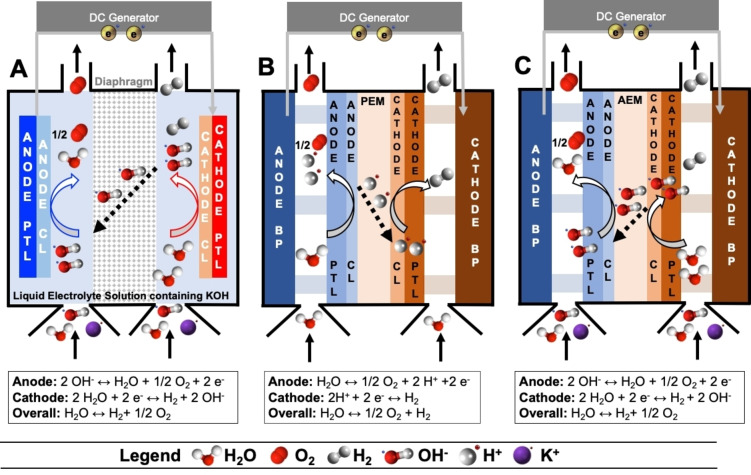
Schematic of (A) A‐WE, (B) PEM‐WE, and (C) AEM‐WE. PEM is the proton‐exchange membrane, AEM is the anion‐exchange membrane, CL is the catalyst layer, PTL is the porous transport layer, BP is the bipolar plate. A‐WE operates with concentrated KOH liquid electrolyte that is recirculated. PEM‐WE operates with pure water. AEM‐WE currently operates with diluted KOH or K_2_CO_3_ with the possibility for the future of operating with pure water while maintaining high performance.

The schematics of the operation of A‐WE and PEM‐WE are shown in Figure 1A,B, respectively. In A‐WEs, the electrodes are dipped in a liquid alkaline electrolyte (highly concentrated KOH) and are separated by a porous diaphragm (e. g., a composite of zirconia and polysulfone).[^30^, ^31^, ^32^, ^33^] A‐WEs are fed with recirculating and/or fresh KOH, to supply the water needed for H_2_ production in the system and to keep the KOH concentration constant. Because of the significant activity of nickel towards HER and OER in alkaline environments, and, alternatively, of stainless steel for OER, the electrodes of A‐WE are often PGM‐free and mainly composed of nickel‐based foam, often doped with Fe,[^34^, ^35^, ^36^] and/or stainless steel.^[37]^ However, PGM electrocatalysts have been sometimes used on both anode and cathode or just on a single electrode to improve the hydrogen production capacity.^[38]^ For anode porous transport layer (PTL), the preferred material in alkaline electrolytes is nickel foam while for the cathode PTL, due to the less extreme conditions, both carbon cloth/paper and nickel foam can be used. In A‐WE, the PTLs contribute only 8 % of the overall stack cost.^[39]^ This is because there is no need for PGM coatings at the anode side due to the alkaline environment. Usually, bipolar plates (BPs) are made of nickel‐coated stainless steel, affecting only 7 % of the entire stack.^[39]^

In contrast to A‐WEs, the electrodes in PEM‐WEs are separated only by a PEM, and the device is fed with deionized water. Despite the water feed, the environment at the electrodes’ interface is highly acidic because of the acidic nature of the PEM and of the cation‐exchange ionomer (Figure 1B).^[40]^ Accordingly, to withstand the low local pH, PEM‐WE electrodes must use PGM‐based electrocatalysts, particularly iridium at the anode and platinum at the cathode. PEM‐WEs do not use carbon black support at the anode to avoid the carbon corrosion that takes place at high potentials.^[40]^ PEM‐WEs employ BPs made of titanium coated with Pt and Au at the anode and cathode, respectively. Because of the extreme potentials and the acidic pH of PEM‐WEs, the anode PTLs are generally made of a titanium felt,^[41]^ often coated with platinum. The Pt coating is needed at the anode in order to minimize titanium oxidation. It was estimated that the Pt loading (due to the PTLs and BPs) exceeds 1 mg cm^−2^, or 0.5 g kW^−1^, which is higher than the typical Pt loading in PEM fuel cells.^[39]^ PGMs other than Pt have also been proposed for improving the durability of the PTLs. Iridium looks particularly promising by decreasing the contact resistances,^[42]^ but it is a very rare element and extremely costly. The conditions at the cathode PTL are less demanding due the low electrochemical potential and absence of water feed on the cathode side. Accordingly, cathode PTLs can be made of porous sintered titanium or carbon cloth, without protective PGM coating. Thus, anode PTLs can contribute up to 17 % to the capital expenditure of PEM‐WEs.^[39]^ Remarkably, this figure of merit is similar to the cost of the entire membrane electrode assembly, which contributes to the stack cost with 24 %.^[39]^ Another important cost for the PEM‐WE originates from the BPs since they are typically covered with a Pt coating to protect them from corrosion during PEMWE operation, especially at the anode.

### 2.2 Advantages and bottlenecks

The state‐of‐the‐art performances of A‐WEs and PEM‐WEs and their near‐future performance envisioned by the Fuel Cells and Hydrogen Joint Undertaking (FCH JU) are summarized in Table 1.^[43]^ These objectives consider the improvements in current density, stability/durability, and hydrogen produced while they envision a reduction in capital and operational costs by approximately one order of magnitude, mainly obtained through a drastic reduction in the use of CRM.

**Table 1:** Comparison of state of the art and future targets according to FCH JU for hydrogen production with A‐WEs and PEM‐WEs.^[43]^

Parameter	State of the art		FCH JU target		
	2012	2017	2020	2024	2030
**A‐WE single‐cell**					
electricity consumption at nominal capacity [kWh kg^−1^]	57	51	50	49	48
capital cost [EUR (kg d^−1^)^−1^] ([EUR kW^−1^])	8000 (≈3000)	1600 (750)	1250 (600)	1000 (480)	800 (400)
operation and maintenance cost [EUR (kg d^−1^)^−1^ yr^−1^]	160	32	26	20	16
**A‐WE stack**					
degradation [% (1000 h)^−1^]	–	0.13	0.12	0.11	0.1
current density [A cm^−2^]	0.3	0.5	0.7	0.7	0.8
use of CRMs as catalysts [mg W^−1^]	8.9	7.3	3.4	2.1	0.7
**PEM‐WE single‐cell**					
electricity consumption at nominal capacity [kWh kg^−1^]	60	58	55	52	50
capital cost [EUR (kg d^−1^)^−1^] ([EUR kW^−1^])	8000 (≈3000)	2900 (1200)	2000 (900)	1500 (700)	1000 (500)
operation and maintenance cost [EUR (kg d^−1^)^−1^ yr^−1^]	160	58	41	30	21
**PEM‐WE stack**					
degradation [% (1000 h)^−1^]	0.375	0.250	0.190	0.125	0.120
current density [A cm^−2^]	1.7	2.0	2.2	2.4	2.5
use of CRMs as PGM electrocatalysts [mg W^−1^]	–	5.0	2.7	1.25	0.4
use of CRMs as catalysts Pt [mg W^−1^]	–	1.0	0.7	0.4	0.1

The overall efficiency and performance of the state‐of‐the‐art A‐WEs are relatively low, with a maximum operating current density of typically 0.5–0.7 A cm^−2^ at 1.7 V.^[39]^ Due to the use of a porous diaphragm to separate the electrodes, A‐WE can directly produce pressurized H_2_ but at relatively low pressure. This is an important limitation, as additional components and energy are required for pressurizing hydrogen. In addition, A‐WE operates with concentrated KOH (≈6 m), posing challenges to materials’ durability and safety.^[44]^ These extreme operating conditions force the utilization of nickel‐based bipolar plates or nickel‐coated stainless‐steel BPs and other system components. The utilization of a porous diaphragm to separate the electrodes poses a safety risk, with high crossover of H_2_ gas and related explosion risk. To reduce this risk, the anode and cathode are typically not in zero‐gap configuration with the diaphragm but separated from it by a layer of liquid electrolyte.^[44]^ This configuration increases the ohmic resistance of the single cell, limiting the current density at which A‐WEs typically operate. These factors together translate into high hydrogen operation production costs, even if the capital cost of A‐WE is attractive.^[43]^ Projects for delivering A‐WEs technology plants up to 100 MW with hydrogen productivity of 41 MT per day are currently in progress. For example, the project GreenH2atlantic has the objective to build such a plant by the end of 2025, showing the viability of the technology at “Technology Readiness Level 8, leveraging scale‐up, standardization and manufacturing automation”.^[45]^

The configuration of anode, membrane, and cathode in a sandwiched membrane electrode assembly (MEA) lowers the ohmic resistance in PEM‐WEs [e. g., area specific resistance (ASR) 68 mΩ cm^2^],^[46]^ allowing them to operate at higher current densities than A‐WEs (>1.5 A cm^−2^). PEM‐WE produces pressurized hydrogen up to 15–50 bar, strongly reducing the energy input and cost, necessary later on for increasing the pressure up to 350–700 bar, the required pressure for vehicles.^[47]^ Thus, PEM‐WEs have relatively low operating costs compared to A‐WEs, but high capital costs because of the use of PGM electrocatalysts and titanium hardware. Another drawback of PEM‐WEs is that the membrane leans on fluorinated polymers. Fluoro chemistry has a strong environmental impact both at the production stage of PEMs (production of tetrafluoroethylene) and at the end of life of PEM‐WE, with no green process existing for recovering the fluorine.[^48^, ^49^] Perfluoroalkyl polymers will probably be banned soon, except for applications where they cannot be substituted.[^8^, ^9^] This aspect might introduce a potential barrier for the deployment of PEM‐WE technology. In contrast to PEMs, most of the developmental AEMs do not use fluorine. Despite of these limitations, PEM‐WE is now considered the most promising electrolyzer technology. A 10 MW plant is operational since July 2021,^[50]^ and a scale up to 100 MW is currently under consideration, with a possible delivery by the end of 2024.

## 3 Anion‐Exchange Membrane Water Electrolyzers

Combining the current advantages of liquid‐electrolyte A‐WEs (low capital cost) with those of the PEM‐WEs (low operating cost, pressurized and ultrapure H_2_, increased safety), AEM‐WEs (Figure 1C) have the potential to operate at significantly reduced cost, without the utilization of CRMs and PGMs in any cell component, with fluorine‐free polymeric membranes, opening new venues for massive and sustainable production of green H_2_ at scale.^[51]^ However, AEM‐WE is still an infant technology when compared to A‐WE and PEM‐WE, leaving a large space for further research and development.^[51]^ Most of the materials used for AEM‐WEs today have been borrowed from PEM‐WEs and/or A‐WEs and are not specifically designed for AEM‐WEs. A holistic approach is urgently needed, not only to improve each material separately but to produce high‐performance AEM‐WE single‐cells based on Earth‐abundant elements.

**Figure 2:**
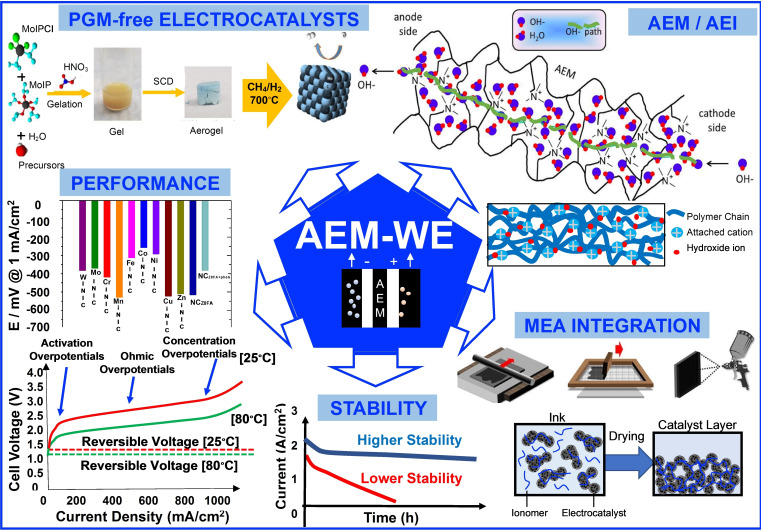
Approaches to improve AEM‐WEs considering PGM‐free electrocatalysts, AEMs/AEIs, MEA integration, performance, and durability/stability. The schematic related to the AEM based on a quaternary ammonium pendant functional groups and with highlighted the OH^−^ path (top right) was adapted from Ref. [54] under CC BY‐NC‐ND 4.0 (https://creativecommons.org/licenses/by‐nc‐nd/4.0/). An example of synthesis schematic (top left) of a PGM‐free electrocatalyst for HER was adapted with permission from Ref. [101]; copyright 2021, American Chemical Society. The figure related to the HER activity for M−N−C and N−C eectrocatalysts at pH 13 (middle left) was adapted from Ref. [102] under CC BY 4.0 (https://creativecommons.org/licenses/by/4.0/).

The rational design and integration of electrode, membrane, and PTLs within the MEA, along with durability studies, will be crucial to develop an AEM‐WE technology with low cost, high performance, high durability, and low environmental impact, as exemplified in Figure 2. Two major drawbacks can be identified, namely the stability and durability of AEMs (and anion‐exchange ionomers, AEIs), and the development of PGM‐free, high‐performance electrodes both for OER and HER. Up to now, AEM‐WE requires the use of dilute KOH or K_2_CO_3_.[^25^, ^52^] However, while PGM‐free active electrocatalysts for OER and HER have been developed in liquid alkaline electrolytes,^[53]^ their implementation in AEM‐WE fed with pure water has hitherto resulted in low cell performance, and additional KOH or K_2_CO_3_ is required.[^25^, ^52^] In the sections below, a brief description of the state‐of‐the‐art AEM/AEI and electrocatalysts and their challenges are presented.

### 3.1 Anion‐exchange membranes

AEMs have been investigated more in fuel cells than in electrolyzers; however, the related features and issues are almost identical. AEMs must allow hydroxide anions to migrate from the cathode to the anode. Differently than A‐WE, where a non‐conducting diaphragm is used, AEMs physically separate the two compartments and avoid crossover of reagents and products. Hence, AEMs must be: (i) ionically conductive; (ii) thermally stable; (iii) mechanically stable; (iv) chemically stable; (v) electrochemically stable, and to make them more attractive, they also need to be low cost, easy to process, and produced by sustainable processes.[^54^, ^55^] Unfortunately, to some extent, a compromise between their mechanical strength and ionic conductivity has to be made. While an increase in the loading of the AEM's functional groups might be advantageous for the hydroxide conductivity, this increases the water uptake, which tends to lower the mechanical stability.^[56]^ In contrast, a lower loading of functional groups might lead to lower ionic conductivity, and therefore a low AEM‐WE performance. It must be underlined that AEMs can achieve very high values of hydroxide conductivity. A hydroxide conductivity as high as 300 mS cm^−1^ was recently reported, exceeding the H^+^ conductivity in PEMs.^[57]^

The most common relevant backbones cited in the literature used for AEMs are: polysulfone,^[58]^ poly(ether ketone),^[59]^ poly(phenylene oxide),^[60]^ fluorinated,^[61]^ polybenzimidazole,^[62]^ polyethylene,^[63]^ polystyrene,^[64]^ polyethylenepyrrole‐*co*‐polyethyleneketone,[^65^, ^66^, ^67^] poly(ethylene‐*co*‐tetrafluoroethylene),^[68]^ and poly(vinylbenzyltrimethylammonium)‐*b*‐poly(methylbutylene) type.^[69]^ Some of these AEM backbones showed excellent performance in AEM‐WE. For instance, poly(fluorenyl aryl piperidinium)‐, polycarbazole‐, and polyphenylene‐based AEMs were reported in cells achieving current densities as high as 2.3, 2.57, and 5.3 A cm^−2^, respectively, measured at 1.8 V, with very good durability.^[52]^ Table 2 summarizes some of the best‐performing AEM‐WEs and their AEM backbones.

**Table 2:** Best‐performing AEM‐WEs identifying the AEM backbones.

Name	Backbone	Functional group	Current density (at 1.8 V) [A cm^−2^]	*T* [°C]	Electrolyte	Ref.
HTMA‐DAPP	polyphenylene	hexamethyl trimethyl ammonium	5.3 3.2 2.7	60 60 85	1 m NaOH 0.1 m NaOH pure H_2_O	[52]
QPC‐TMA	poly(carbazole)	poly(9‐(6‐(trimethylammonium bromide)hexyl)‐9*H*‐carbazole)	2.57	70	1 m KOH	[70]
*m*‐PBI	polybenzimidazole	poly(2,2′‐(*m*‐phenylene)‐5,5′‐ bibenzimidazole)	1.7	8	24 wt% KOH	[71]
PFTP	poly(fluorenyl aryl piperidinium)	piperidinium	2.3	80	1 m KOH	[72]

The hydroxide ion transport is assured by N‐containing, positively charged groups (e. g., ammonium,^[73]^ piperidinium,^[74]^ spirocyclic^[60]^ isoindolinium,^[75]^ imidazolium,[^76^, ^77^, ^78^] etc.). Cations others than N‐based were also used, such as phosphonium,^[59]^ sulfonium,^[79]^ and metal‐containing anion‐conducting groups, such as complexes of Ru^II^,^[79]^ cobaltocenium,^[79]^ Ni^II^,^[80]^ and Au^II^,^[81]^ have been described. Alternative cationic groups were also exploited, such as guanidinium^[82]^ and carbazolium.^[83]^

Few AEMs are to‐date commercially available from companies, such as Tokuyama (A201) (discontinued production), Fumatech (Fumasep®), Ionomr (Aemion^TM^), Dioxide Materials (SUSTAINION®), and Orion Polymer (Orion TM1).^[28]^ Indeed, there are research‐based membranes, for example, by Versogen (PiperION)^[84]^ and Xergy (XION™ Ion Exchange Membranes),^[85]^ which are expected to outperform commercial membranes and their systems. However, they are available on the market but still very expensive due to their low production scale, and at the current stage they are not cost‐competitive with commercial PEMs. Moreover, it is not yet clear if they fulfill the durability requirement under harsh conditions in the electrolysis cell.

**Figure 3:**
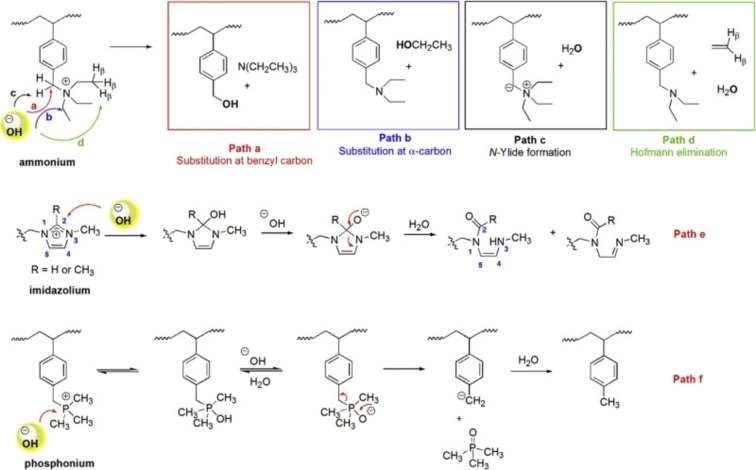
Identified degradation routes of different cationic functional groups: benzyltrimethylammonium, imidazolium, and phosphonium. Adapted with permission from Ref. [86]; copyright 2018, Elsevier.

One of the biggest challenges in the development of AEMs/AEIs is to overcome their high alkaline degradation. Developing highly stable cationic groups to increase AEM stability is crucial to allow them to operate in the aggressive environment of AEMs. It is well known that the hydroxide anions attack the positively charged cations in the AEM, neutralizing them and suppressing their conductivity. For the cationic functional groups, the main identified mechanisms of degradation reported in the literature are Hofmann elimination (E2), S_N_2, and N‐ylide formation, although other mechanisms were also investigated and observed in different functional groups, such as single electron transfer (SET), deprotonation, ring‐opening, and benzyne mechanisms.[^62^, ^83^, ^86^, ^87^, ^88^, ^89^, ^90^, ^91^] In Figure 3, as example, the degradation mechanisms of benzyltrimethylammonium (BTMA), imidazolium, and phosphonium are reported. The different paths of degradation are described in detail in Ref [86].

In recent years, several new quaternary ammonium salts have been proposed to address this challenge,^[92]^ but while they perform well in ex‐situ chemical studies, their performance is very limited under normal operating conditions in electrolyzer cells. Two quaternary ammonium (QA) salts were reported to be reasonably stable in alkaline media: trimethylbenzylammonium (TMBA) and 6‐azonia‐spiro[5,5]undecane (ASU), both of which have been shown to react with hydroxide ions in reasonable times. TMBA typically reacts with hydroxide through an S_N_2 reaction mechanism (nucleophilic hydroxide attacks an electrophilic carbon), while ASU reacts through an E2 mechanism (hydroxide acts as a base, abstracting a proton). TMBA was largely investigated, and its stability was also found to strongly depend on the hydration level.^[91]^ Given that TMBA decomposes by the attack of the hydroxide at the benzylic position, further stabilization of the molecule by increasing the transition state energy for this nucleophilic attack is required. Solutions might be envisioned in the change of the steric environment around the benzylic carbon or by the increase of the electron density at this carbon.^[90]^

Taken all together, it should be highlighted that the most important parameter responsible for the instabilities and low conductivity of the AEM is the degree of hydroxide dissociation in polar side chains. Indeed, an effective dissociation of hydroxide acts to improve AEMs’ ionic conductivity, chemical stability (the chemical attack to side groups with their concurrent degradation is inhibited), and thus operating temperature range Δ*T*=*T*_δ_−*T*_g_, by lowering the glass transition temperature (*T*_g_) and raising the temperature of the disorder‐order transition (*T*_δ_).[^69^, ^93^]

### 3.2 Route for efficient anion‐exchange membrane and ionomers

Despite gigantic improvements that have been achieved in developing AEMs and related ionomers, important gaps still have to be filled. Stability has to be improved significantly. Chemical stability has to be enhanced, avoiding the deterioration of the functional groups because of the attack of hydroxide. This can be obtained through a careful choice of the starting polymeric matrix and of the functional groups used to ensure anionic transport. Aid in this sense can come from modeling [e. g., density functional theory (DFT)]. Mechanical stability has to be upgraded in order to allow the operation at higher pressure, obtaining pressurized hydrogen at higher pressure, which is beneficial for the final use. This can be obtained by cross‐linking and/or use of inorganic fillers. Thermal stability needs to be amended in order to allow the operation of AEM‐WE at higher temperature (up to 90 °C), which in turn would improve the electrodes kinetics and AEM conductivity. The AEM must also play the role of a physical separator, to minimize the crossover of gases from one compartment to another. Despite already reported high hydroxide conductivity values, the AEM ionic conductivity can be further enhanced while keeping the stability also high. In recent Project Calls from the FCH JU, the target values for AEM were (i) ASR lower than 70 mΩ cm^2^ and (ii) ion conductivity greater than 50 mS cm^−1^.^[28]^

Importantly, the thickness of the AEM should be minimized in order to lower the ohmic resistance. This, however, must be achieved without compromising the mechanical stability of the AEM. An interesting route, in this sense, could come from the utilization of micropatterning and nanopatterning using lithographic techniques. AEIs must assure good chemical compatibility with the membranes, and the ion exchange capacity (IEC) values must be carefully tuned to minimize mass transport overpotentials.

Notably, AEM‐WEs should operate with water instead of dilute KOH or K_2_CO_3_, in order to diminish the capital, operational, and maintenance costs of AEM‐WEs. As deionized water is expensive, lower‐quality water (containing a low quantity of dissolved ions) should be used. Therefore, the AEM should be resilient to impurities for improved durability. Importantly, the development of low‐cost and fluorine‐free AEM is paramount for the massive deployment of AEM‐WEs as an environmental and economical sustainable technology. Fundamental studies have to be conducted to increase the understanding of the OH^−^ transport phenomena, identifying and mitigating degradation processing occurring in operando conditions.

## 4 PGM‐Free Electrocatalysts

**Figure 4:**
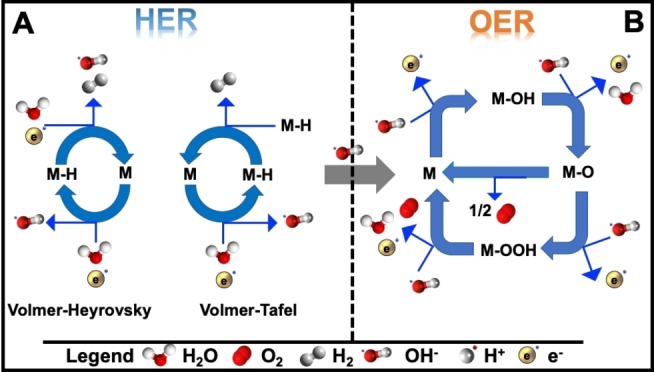
Schematic of the (A) HER and (B) OER reaction steps in alkaline environment.

HER and OER are the two main reactions occurring in water electrolyzers. In alkaline media, the hydrogen evolution occurs following two steps. The first one, named as Volmer step, consists in the dissociation of the water molecule into hydroxide and the formation of the hydrogen intermediates (H_ad_). The second step can occur electrochemically following the Heyrovsky step or through a chemical recombination (Tafel step).^[94]^ The schematic illustration of the HER steps is drawn in Figure 4A.

In alkaline environment, OER occurs following different steps. In the first step, M−OH is formed through a one‐electron oxidation of hydroxide adsorbed on the active site. The second step is the transformation of M−OH into M−O with two protons and electrons removed. In the third step, M−O will follow two different pathways to form O_2_ molecules. In one case, two M−O species will combine and convert the intermediates into O_2_, bringing back the M active sites at their initial state. In the other case, M−O will couple with a hydroxide forming M−OOH through a one‐electron oxidation. In the latter, the last step will involve another coupling with hydroxide generating O_2_ and water, and the M active sites will return to their initial state.[^95^, ^96^] The schematic illustration of the OER steps is drawn in Figure 4B.

Having in principle a high pH environment (with AEM and AEI in their OH‐form, even if no liquid alkaline electrolyte is used), AEM‐WE can allow the substitution of PGM electrocatalysts with Earth‐abundant first‐row transition metals (e. g., Mn, Fe, Ni, Cu, and Mo) at both the anode and cathode for catalyzing the OER and HER, respectively. Specifically, in using AEM‐WE, Ir (≈$5200 oz^−1^) and Pt (≈$950 oz^−1^) could be completely substituted using Fe, Ni, Mn, Cu, and Mo, significantly reducing the costs of the overall system.

**Figure 5:**
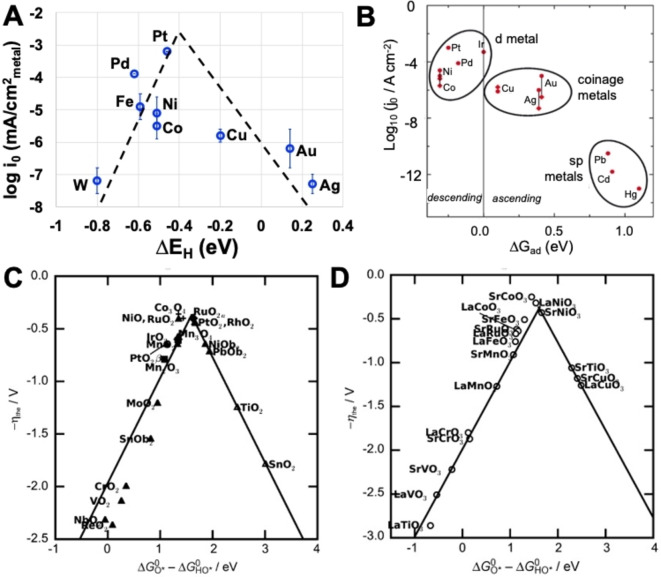
(A) Exchange current densities [log(*i*_0_)] on monometallic surfaces as a function of the calculated hydrogen binding energy. (B) Volcano curve for HER electrocatalysts at various metals in function of the energy of adsorption Δ*G*_ad_. OER theoretical overpotential vs. the differences between the standard free energy of two subsequent intermediates Δ*G* ^0^_O*_−Δ*G* ^0^_HO_ considering (C) various binary oxides and (D) perovskite oxide. (A) Reproduced with permission from Ref. [97];copyright 2013, Royal Society of Chemistry. (B) Adapted from Ref. [98] under CC BY 4.0 (https://creativecommons.org/licenses/by/4.0/). (C,D) Adapted with permission from Ref. [99]; copyright 2011, Wiley‐VCH.

In HER, the selection of the transition metals can be evaluated starting from the Volcano plots (Figure 5A,B).[^97^, ^98^, ^99^] In this case, the Volcano plots show the exchange current densities plotted as a function of the hydrogen binding energy (HBE) calculated by DFT. In this plot, the top of the Volcano is occupied by metals capable of adsorbing hydrogen neither too strongly nor too weakly. These metals are PGMs, possessing also the best electrocatalytic activity towards HER. As suggested by Abbasi et al.,^[27]^ coupling two or more first‐row transition metals into alloys could have beneficial and synergistic effect to climb the Volcano by improving their hydrogen adsorption capability till reaching electrocatalytic activity similar to the one of PGMs.

While the Volcano plot for HER considers only pure metallic surface and is therefore far from that of applied catalysts and applicative conditions, its use can facilitate the selection of metals during the synthetic process. One interesting route that could be exploited would consider the combination of one metal standing in the ascending and another one from the descending section of the Volcano plot, in order to optimize the HBE in bimetallic electrocatalysts. In parallel, for HER electrocatalyst, carbon‐supported electrocatalysts containing active sites are generally used. Therefore, the research has to be dedicated also to the selection as well as to the development of conductive and high‐surface‐area carbonaceous support on top of which metallic active sites have to be dispersed. Conductive carbonaceous support also plays a crucial role in the electrocatalytic activity of HER electrocatalysts.^[27]^

Single transition metal, bimetallic, and trimetallic electrocatalysts were synthesized starting from metal‐organic frameworks (MOFs), covalent organic frameworks (COFs), transition metal salts, and metal‐containing phthalocyanines or porphyrins in order to synthesize transition metal phosphides, chalcogenides, carbides, or M−N−C‐type electrocatalyst.[^17^, ^100^, ^101^, ^102^, ^103^, ^104^] Alternative electrocatalysts following biomimicking or bioinspired routes containing Ni−Fe or MoS_2_ were synthesized and characterized, showing promising performance.[^105^, ^106^, ^107^, ^108^, ^109^, ^110^] The interplay between surface chemistry and surface morphology is required. Importantly, an HER electrocatalyst should possess an ultra‐high number of active sites and ultra‐high catalyst site utilization while supported on carbonaceous backbone surface. In parallel, the electrocatalyst should have a well‐defined hierarchical structure with a high surface area and designed porosity to enhance the release of hydrogen gas formed in the micropores.

Similarly to HER, the Volcano plot for OER operating in alkaline environment is presented (Figure 5C,D). In OER, because of the high electrochemical potential at the anode side, corrosion can be detrimental, and electrocatalysts cannot be supported on carbonaceous materials because they suffer from very low durability, mainly for carbon corrosion. The shown Volcano plot for OER electrocatalysts does not apply to single‐metal atom sites but it considers transition‐metal oxides and perovskites. The Volcano plots for OER represented in Figure 5C,D use as indicator for the catalytic activity the difference between the energy states of two subsequent intermediates (Δ*G*_OHOO*_−Δ*G*_OHO*_).^[99]^ In the case of weak oxygen adsorption on the surfaces, the potential is limited by the oxidation of HO* due to the fact that intermediates cannot easily react. In contrast, in the case of strong oxygen adsorption on the surfaces, the potential is limited by the formation of HOO* species, the intermediate states, and the adsorbed products are stable.^[111]^

Therefore, carbides (e. g., Mo_2_C), spinels, oxides, perovskites, and ceramic materials are the most promising PGM‐free electrocatalysts considering the tradeoff between performance and durability.[^111^, ^112^, ^113^, ^114^, ^115^, ^116^, ^117^, ^118^] Sub‐stoichiometric metal oxides, carbides, and nitrides seems to be excellent electrocatalysts as they are expected to keep their structure even under high oxidative potentials. In addition, core–shell structures with conductive cores are suitable, allowing good electronic conductivity while protecting the core from oxidation. Most robust OER electrocatalysts are metal oxides, which make them corrosion‐resistant, but that comes with the price of decreased conductivity; therefore, a trade‐off is expected. It has been shown that a thin passivation layer can protect from further corrosion while not lowering the conductivity significantly. Often, cobalt is used in bimetallic spinels and oxides, but it should be avoided as listed in the EU CRM list.^[6]^ Similar to HER electrocatalysts, the addition of other elements might change the electronic structure and provoke the alteration on the binding energy of the intermediates, therefore leading to higher electrocatalytic activity by climbing the Volcano plot. Moreover, OER electrocatalysts must have a high number of active sites and proper morphology, favoring degassing.

As it seems, the realization of PGM‐free electrocatalysts becomes a reality with Ni‐based OER electrocatalysts in AEM‐WEs, which currently are considered as the benchmark OER electrocatalysts.[^121^, ^122^, ^123^] In contrast, for the HER, the state‐of‐the‐art electrocatalysts are still based on precious metals. Some of the best HER and OER electrocatalysts today and their performance are presented in Table 3.[^119^, ^120^, ^121^, ^122^, ^123^]

**Table 3:** Examples of highly active HER and OER electrocatalysts. *η* is the overpotential and *j*_η_ is the current density where the overpotentials are measured.

Electrocatalyst	Reaction	Electrolyte (KOH)	*η* [mV]	*j*_η_ [mA cm^−2^]	Tafel slope [mV dec^−1^]	Ref
PtNi(N) NW	HER	1 m	13	10	29	[119]
PtNi−O/C	HER	1 m	39.8	10	78.8	[120]
NiFeTiOOH	OER	0.1 m	400	10	–	[121]
Ni_0.83_Fe_0.17_(OH)_2_	OER	1 m	245	10	61	[122]
NiFe‐LDH NPs	OER	0.1 m	151	30	50	[123]

It has become common to use the overpotential measured at 10 mA cm^−2^ as a catalytic performance descriptor for both OER and HER. In parallel, also Tafel slopes can be used for comparison among different electrocatalysts leading to the selection of the electrocatalyst. However, both methods are not accurate descriptors and open to interpretation. Most of the studies of these electrocatalysts are conducted in liquid electrolyte electrochemical cells, and testing in electrolyzers is needed to confirm these results under realistic operating conditions. To‐date, durability studies are not conducted according to a standardized protocol, and thus durability results are hard to compare between studies. One technical barrier to the development of OER and HER electrocatalysts is the lack of proper accelerated stress tests to study their stability and durability.

It is important to note that the lack of formal or standardized performance descriptor, from one or more of the research agencies such as the US‐DOE or EU Programs, which includes the electrocatalyst mass activity, reaction turnover frequency, electrochemically active surface area, or site density, measured at certain experimental conditions. These descriptors are necessary for the advancement of the field.

To the best of our knowledge, only one company (Pajarito Powder LLC, USA) is currently selling commercially PGM‐free HER electrocatalysts.^[124]^

While the development of novel CRM‐free HER and OER electrocatalysts specifically designed for high‐pH environment is critically needed, their rational integration in the MEA of an AEM‐WE fed and operating with pure water will likely be as critical. As an example, the implementation at the anode side of PGM‐free OER electrocatalysts known to be highly active in liquid alkaline environment has hitherto resulted in low AEM‐WE performance.[^116^, ^125^, ^126^, ^127^]

**Figure 6:**
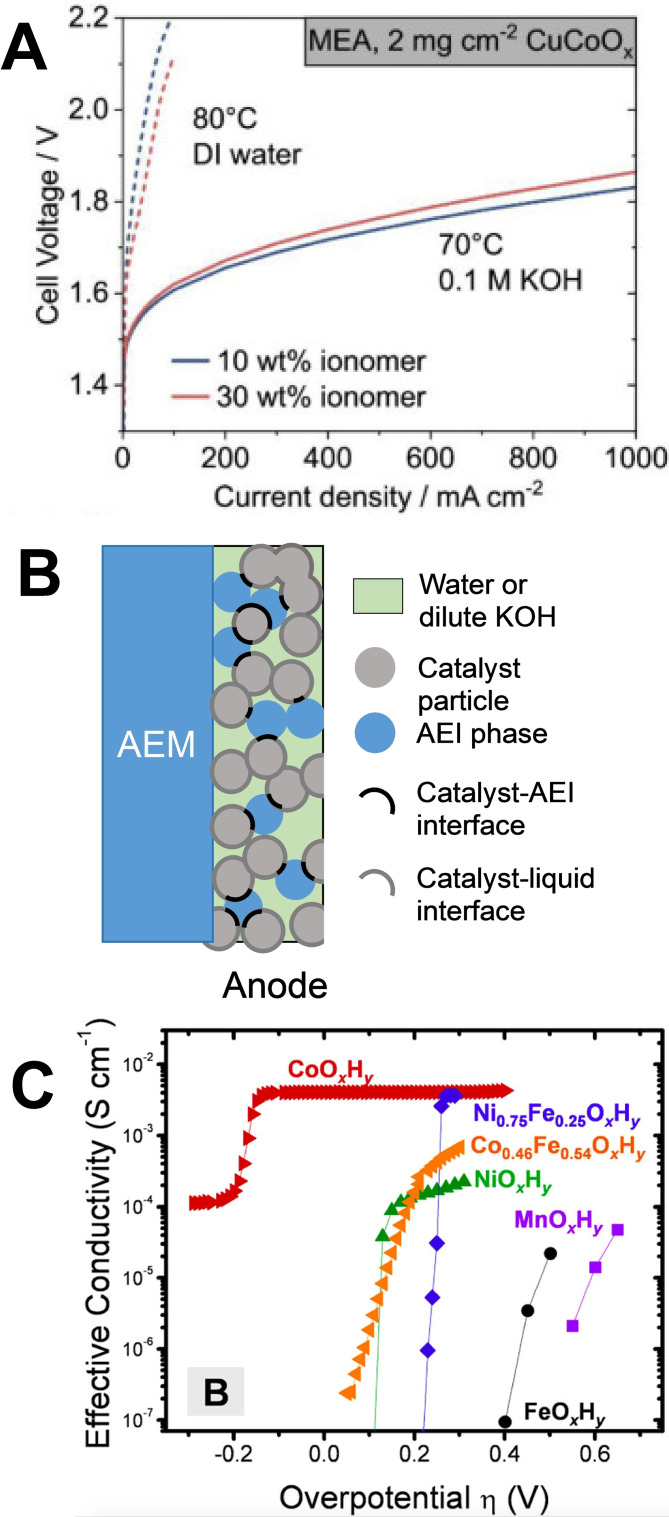
(A) Effect of anolyte feed on the AEM‐WE performance for a CuCoO_*x*_ anode and with otherwise same operating conditions, cathode, and electrode preparation. (B) Scheme of an anode structure composed mainly of a catalyst–liquid interface and with a minor presence of catalyst‐AEI interface. (C) Effect of the electrochemical potential on the electronic conductivity of thin‐film oxyhydroxide films in 1 m KOH. (A) Adapted from Ref. [125] under CC BY 4.0 (https://creativecommons.org/licenses/by/4.0/). (C) Reproduced with permission from Ref. [131]; copyright 2015, American Chemical Society.

Several studies concluded that a dilute KOH feed (0.01–1.0 m KOH) is critical to reaching a high performance with PGM‐free anodes in AEM‐WE.[^52^, ^125^, ^127^, ^128^, ^129^] Figure 6A is an example of the effect of the anolyte feed nature on the AEM‐WE performance, in otherwise fixed conditions and for a same MEA, comprising a CuCoO_*x*_ anode. The effect is huge and can be explained by increased OER overpotential on CuCoO_*x*_ at neutral pH compared to high pH, and also increased cell ohmic resistance.^[125]^ The same trends were observed with IrO_2_ anodes, the switch from 1 m KOH to pure water feeding leads to an increase in cell voltage of 300–400 mV at current densities of 0.4–1.0 A cm^−2^.^[130]^ Similar behavior was detected by switching the electrolyte from 1 m NaOH to pure water.^[52]^

While the use of dilute KOH in AEM‐WE instead of concentrated KOH in A‐WE is a step forward, it still brings more complexities to the system compared to PEM‐WE, and also has drawbacks regarding the H_2_ purity. The data seems to show that both PGM and PGM‐free anode electrocatalysts are not in direct contact with the AEI (associated with high local pH) during operation of AEM‐WE but are instead indirectly connected to the AEI network via the liquid. Thus, when pure water is fed to the anode, most of the electrocatalytic area comprises an electrocatalyst–water interface (represented by the dark grey outlines in Figure 6B), with a minor fraction of the electrocatalytic area consisting of a true AEI‐electrocatalyst interface (represented by the black arcs in Figure 6B). The neutral pH of water leads to several drawbacks, such as high ohmic drop through the anode layer, increased OER overpotential compared to high pH conditions, and decreased thermodynamic stability for many metal oxides, and in particular for PGM‐free metal oxides.[^125^, ^128^, ^130^] This view of the anode being composed mainly of an electrocatalyst–liquid interface and with a minor contribution from the catalyst‐AEI interface was recently supported by different stability behavior with deionized water feed than with 0.1 m KOH feed on a CuCoO_*x*_ anode, showing that the change in initial performance is not only due to a lower electrochemical area with a water feed (as might have been the case, if water leads, e. g., to different wetting properties than dilute KOH), but is due to a different pH experienced by most of the electrocatalytic surface.^[125]^ Further increasing the complexity of AEM‐WE PGM‐free anode understanding and development, the electrical conductivity of (oxy)hydroxide thin films of the first row of transition metals was shown to drastically depend on the applied electrochemical potential in 1 m KOH, with a clear switch from non‐conductive to conductive properties at positive overpotentials for OER, except for CoO_*x*_H_*y*_ (Figure 6C).^[131]^ The electrical conductivity can thus change drastically with potential and may also change drastically from KOH to pure water electrolyte, since cation intercalation from the electrolyte also can impact the structure and electronic conductivity of (oxy)hydroxide thin films. Fundamental understanding of the catalyst–AEI and electrocatalyst–electrolyte interface is therefore still needed for already known and also for novel CRM‐free OER electrocatalysts, with a combination of mathematical modeling, methods, and diagnostics to understand how different AEI and different CRM‐free electrocatalysts are spatially organized in anodes, and how this impacts the local pH at the electrochemical interface, as well as the anode's ionic and electronic conductivities, and finally, its performance. The same challenges and questions may also apply, at least in part, to the development of PGM‐ and CRM‐free anodes.

### 4.1 Route for efficient electrocatalysts

Because of the operation at high pH, PGM‐free electrocatalysts composed of earth‐abundant first‐row transition metals should be used for both anodic and cathodic reactions. Cobalt, which is identified as a CRM by the EU,^[6]^ should be replaced with a non‐CRM. Bi‐ or trimetallic electrocatalysts seem to be the most promising choice leading to the top of the Volcano plot.

Up to now there is no official or scientifically recognized protocol for testing HER and OER electrocatalysts. In fact, very often, the electrocatalytic activity of HER and OER is limited to rotating disk electrode (RDE) technique or electrochemical cell where the current at 10 mA cm^−2^ is selected as being representative of the activity. In many cases, durability tests are conducted by cycling the electrocatalysts in a potential range for thousands of cycles and measuring the increase in the overpotential over time. However, in the majority of the cases, this does not translate into effective and efficient electrocatalytic activity once the electrocatalyst is integrated into the MEA and tested in a full cell electrolyzer. Very recently, half‐cell designs consisting of the electrode in a gas diffusion layer (porous transport layer) configuration were developed and introduced for exploring the electrocatalysts performance in a more realistic way.[^132^, ^133^, ^134^, ^135^, ^136^, ^137^, ^138^] This method is still not ideal since the presence of liquid in a water electrolyzer does not truly mimic the situation of an AEM‐WE fed with water, but the advantage of achieving higher current density can be recognized. The half‐cell method was introduced mainly for other reactions, its utilization for HER and OER is strongly encouraged and supported. This method could help speed up the screening of electrocatalysts in an electrode design and test conditions closer to those in an AEM‐WE than the RDE, likely leading to results more predictive of the materials’ behavior in AEM‐WE. However, ultimately testing novel materials in an electrolyzer will remain necessary, even though it is complex and time‐consuming. The durability of the electrocatalyst has to be fully studied, and the degradation mechanisms have to be understood and consequently mitigated. So far, only a few studies have explored long‐term durability, leaving open interpretation and questions about the reliability of the materials. Importantly, the electrocatalyst has to be synthesized, increasing the number of available active sites and improving their accessibility to the reagents. Starting precursors and thermal treatments must be optimized to synthesize efficient electrocatalysts for both HER and OER. As OER occurs in oxidative conditions where carbon corrosion takes place, a carbon‐supported electrocatalyst is undesired. Lowering the oxidative state of the anode electrode is a must. In contrast, HER can use carbon‐supported electrocatalysts. The morphology of the electrocatalysts also plays an important role, and synthesis processes should also consider this crucial feature. Differently from fuel cell operations, degassing of hydrogen is a limiting step, and the morphology of the electrocatalyst layer must be optimized. Concurrently, the effect of the metal hydroxide content on the performance of HER and OER electrocatalysts should be fully studied and understood.

## 5 Porous Transport Media and Bipolar Plates: State of the Art and Directions

In water electrolysis, hydrogen and oxygen evolution are heterogeneous reactions that occur at the surface of solid electrocatalysts in a liquid medium with the release of gaseous products. The transport of reactants and products to and from the electrocatalyst surface is therefore complex and critical for electrolyzer performance. In membrane‐based electrolyzers, the electrocatalyst faces the membrane on one side and the porous transport layer on the opposite side. The function of the porous transport layer is to provide optimized mass transport and to transport the electrons between the catalyst layer and the bipolar plates.^[139]^ The PTL may have thickness with above 1 mm to support the membrane, enabling the system to withstand high differential pressure, which in turn is required for several applications (e. g., automotive).^[140]^ Accordingly, the PTL must have an engineered porosity and be realized in a material that provides metallic conductivity.

The preferred material for anode PTL in AEM‐WE is nickel. Indeed, the Pourbaix diagram of nickel shows that above 0 V vs. the reversible hydrogen electrode (RHE), the oxidation products are oxides or hydroxides that passivate the surface.^[141]^ In AEM‐WEs, the anode PTLs are usually nickel foams, while cathodes can be nickel foams or carbon cloths. A recent study has shown that the PTL is a major contributor to the performance of AEM‐WEs. In a very recent work,^[142]^ the use of anode PTL has been compared along with the deposition of electrocatalysts directly onto the PTL. It was found that nickel foams are excellent PTLs and can perform better even than platinized titanium. In Ref. [143], stainless‐steel PTLs have been employed, showing that the diameter of the fibers of the stainless‐steel PTL has a remarkable effect on the performance of the AEM‐WE. To date, PTLs are still under the spotlight of research as their optimization has a huge potential for improving the performance of AEM‐WEs. However, in terms of PTL, AEM‐WEs have a double advantage over PEM‐Wes: a significant cut in cost, and a dramatic reduction of the impact on CRMs. Future direction should be dedicated towards the optimization of morphology and porosity in order to enhance degassing without compromising conductivity and mechanical structure. The reduction of PTL thickness could be further pursued in order to decrease the overall cost; once again, this route should not affect the mechanical stability. Substitution of nickel with stainless steel might also lead to a reduction of costs although more durability tests should be pursued in order to strengthen its reliability.

BPs are inherited from A‐WE technologies and are composed by nickel‐coated stainless steel.^[26]^ However, as the direction is to operate AEM‐WE fed with pure water, BPs with lower cost and cheaper materials could be designed and used.

## 6 Membrane Electrode Assembly

The integration of the electrocatalyst in the catalytic layer and in the MEA is critical for performance and durability improvements, but also critical to allow switching from dilute KOH or K_2_CO_3_ to water feed in the near future. Nowadays, MEA fabrication occurs through deposition, coating, or embedded electrode methods, and these are just some of the options that have been investigated and completely inherited from more the mature PEM‐WE technology.^[144]^ In the current status of MEA fabrication, the appropriate combinations of electrocatalyst, ionomer, and membrane must be studied and optimized in order to improve the ionic conductivity through the electrodes. While this option might be considered for diluted KOH or K_2_CO_3_ operation, alternatives need to be adopted for operation in pure water, which is a difficult challenge to overcome.

Different or optimized methods to interface various AEI and electrocatalysts should also be explored, in order to control and improve the interaction and interconnection between AEIs and electrocatalysts. It is critical to keep in mind that not only the chemistries, but also the current AEI morphologies are different compared to those of the cation exchange ionomers (e. g., Nafion ionomer). Therefore, diverse design of the catalytic layer should be pursued and engineered in order to achieve better OH^−^ access to electrocatalyst surface. An intriguing possibility might be related to the engineering of AEI particles, controlling their shape and size in order to optimize the interaction as a function of the nature and morphology of HER and OER electrocatalysts. Another solution might be the creation of a self‐standing three‐dimensional (3D) domain of AEI in which the electrocatalyst is integrated in a following step. Importantly, characterization methods are needed to better understand the nature and extent of the electrocatalyst–AEI interface.

Mechanical issues related to the delamination or dissolution of the catalytic layer must be solved. The AEI combined within the electrocatalytic layer still poses a few unresolved questions related to its role in the anodic and cathodic electrocatalytic process. Other components, such as the PTL, should be optimized to be resistant to corrosion, favoring the degassing and possessing reduced interface resistance between the electrocatalytic layer and the PTL.

## 7 Path to Successful Deployment of AEM‐WE

In our view, AEM‐WE could play a crucial role as a technology for producing green hydrogen. Among the different electrolyzers technologies, AEM‐WE is the youngest in terms of investment, research, and development, and therefore prone to the greatest improvements in the near future. Importantly, AEM‐WE would work with PGM‐free electrocatalysts and fluorine‐free polymeric membranes/ionomers, leading to lower long‐term environmental impacts and a sustainable route for the production of green hydrogen. However, many bottlenecks have to be resolved, especially concerning material stability and durability (membranes/ionomers, electrocatalysts) and their integration into MEAs. The stability and durability of membranes becomes even more challenging and also important to achieve for operating AEM‐We at higher temperature, which would benefit the ionic conductivity and reaction kinetics, leading to an overall higher efficiency and higher hydrogen production rate. The activities proposed for fulfilling the existing gaps were summarized in the International Renewable Energy Agency (IRENA) report.^[39]^ However these proposed activities have been expanded and detailed in Table 4, identifying the effort of the challenge, the benefits that could lead to overcoming the challenges, and the related achievements.

**Table 4:** Proposed activities to overcome limitations identifying the challenge, related benefits, and achievements.

Activitiy	Challenge	Benefits	Achievement(s)
improve AEM chemical stability	difficult	high	improved durability
improve AEM thermal stability	difficult	high	improved durability improved AEM‐WE efficiency
improve AEM mechanical stability	moderate	high	improved durability ability to operate at higher pressure
decrease AEM thickness	moderate	medium	lower ohmic resistance as drawbacks, operational pressure might be reduced and H_2_ purity might decrease
improve membrane selectivity of the reaction products	moderate	moderate	improved H_2_ purity improved membrane durability
improve HER electrocatalysis	moderate	high	improved AEM‐WE efficiency improved electrocatalyst kinetics improved electrocatalyst durability
improve OER electrocatalysis	moderate	high	improved AEM‐WE efficiency improved electrocatalyst kinetics improved electrocatalyst durability avoiding electrocatalyst corrosion
improve electrocatalyst–AEM interface in the MEA	difficult	high	improved durability improved AEM‐WE efficiency lower overpotentials lower ohmic and transport resistance allow feeding with pure water
improve electrocatalytic layer stability	difficult	high	improved durability of the catalytic layer improved AEM‐WE efficiency lower overpotentials
improve MEA–PTL interface	difficult	high	improved durability improved AEM‐WE efficiency improved hydrogen and oxygen degassing

## 8 Outlook

In order to be competitive with the existing electrolyzer technologies, anion‐exchange membrane water electrolyzers (AEM‐WEs) need disrupting materials and technological breakthroughs in electrocatalysts, membranes, ionomers, and membrane electrode assembly (MEA) integrations and operations. The techno‐economical goals to be achieved by 2050 are shown in Table 5.^[39]^ In order to be competitive, AEM‐WEs would need to operate at current densities above 2 A cm^−2^ with an operating voltage lower than 2 V. Advancement in AEM would lead to a higher load range expected to vary from 5 to 200 %, improved system pressure expected to reach 70 bar, and higher operating temperature (80 °C). Higher operating temperature would improve electrocatalytic activity, voltage efficiency (>75 %), and electrical efficiency (42 kWh kg^−1^ H_2_ for the stack and 45 kWh kg^−1^ H_2_ for the overall system). Up to now, the lifetime of the AEM‐WE stack is around 5000 h, but it is expected to skyrocket to at least 100000 h to be competitive. With improvements in electrocatalysts, AEMs and anion‐exchange ionomers (AEIs), and their integration in MEAs, electrode areas are expected to increase sensibly, allowing the AEM‐WE device to reach MW size. While cost has not been yet fully identified as AEM‐WE is a relatively young technology, considering the deployment at MW scale, in order to be competitive, the technology should have a cost of <100 USD kW^−1^ for the stack and <200 USD kW^−1^ for the complete AEM‐WE system.

**Table 5:** State of the art and future key indicators of performance for AEM‐WE technology. Data based on IRENA analysis.^[39]^

Indicator	2020	Target 2050	R&D focus
normal current density	0.2–2 A cm^−2^	>2 A cm^−2^	membrane; electrocatalysts
voltage range (limits)	1.4–2.0 V	<2 V	electrocatalysts
Operating temperature	40–60 °C	80 °C	membrane; electrocatalysts
cell pressure	<35 bar	>70 bar	membrane
load range	5–100 %	5–200 %	membrane
H_2_ purity	99.9–99.999 %	>99.9999 %	membrane
voltage efficiency (LHV)	52–67 %	>75 %	electrocatalysts
electrical efficiency (stack)	51.5–66 kWh kg^−1^ H_2_	<42 kWh kg^−1^ H_2_	electrocatalyst/membrane
electrical efficiency (system)	57–69 kWh kg^−1^ H_2_	<45 kWh kg^−1^ H_2_	balance of plant
lifetime (stack)	>5000 h	100000 h	membranes/electrodes
stack unit size	2.5 kW	2 MW	MEA
electrode area	<300 cm^2^	300 cm^2^	MEA
cold start (to nominal load)	<20 min	<5 min	Insulation (design)
capital cost stack (minimum 1 MW)	unknown	<100 USD kW^−1^	MEA
capital cost system(minimum 10 MW)	unknown	<200 USD kW^−1^	rectifier

## Conflict of interest

The authors declare no conflict of interest.

## Biographical Information

*Carlo Santoro got his Ph.D. at the University of Connecticut in 2009, working on microbial fuel cells. He moved to the University of New Mexico in 2013 working on platinum‐free electrocatalysts for oxygen reduction reaction and supercapacitive bio‐electrochemical systems. Following a spell as Lecturer at the University of Manchester (2020), he joined the University of Milano‐Bicocca in 2021 as Assistant Professor, where he established the Electrocatalysis and Bioelectrocatalysis Lab (EBLab). His work focuses on development of electrocatalysts based on platinum‐group metal‐free materials for electrochemical systems, pursuing biomimetic and bioinspired approaches. He has published over 100 manuscripts and holds 2 patents*.

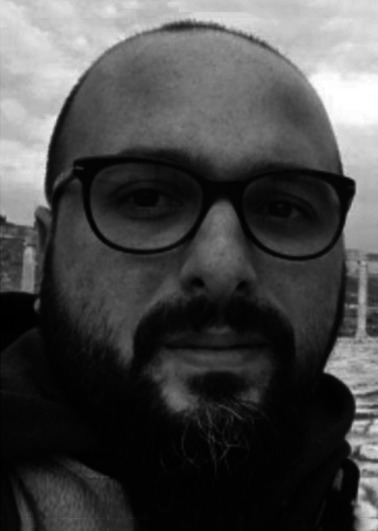

## Biographical Information

*Lior Elbaz is an Associate Professor for Electrocatalysis and Sustainable Energy in the Department of Chemistry, Bar‐Ilan University, Israel. He is the head of the Israeli Fuel Cells Consortium (IFCC), composed of 12 leading Israeli labs, the Israeli representative to the International Energy Agency's Advanced Fuel Cells Executive Committee, and a member of the Israeli Presidential Climate Forum. He is the co‐founder of three Israeli start‐up companies, developing reversible fuel cells, fuel cells for aviation, and fuel cells for stationary power supply. He co‐authored 3 patents and published more than 50 peer‐reviewed research papers in high‐impact journals*.

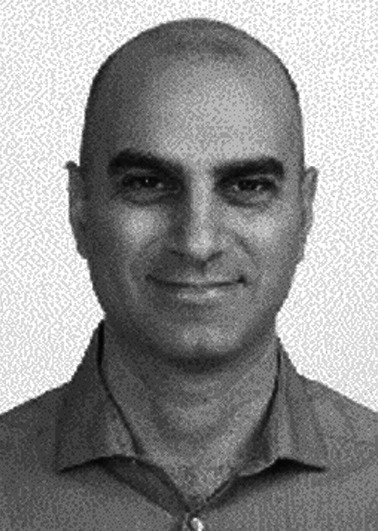

## Biographical Information

*Dario Dekel currently leads a large research group entirely devoted to developing AEM‐based electrochemical technologies. His group studies and develops materials, components, and processes for AEMFCs and AEMWEs, including AEMs, PGM‐free electrocatalysts for hydrogen (and other fuels) oxidation and oxygen reduction reactions, ionomeric materials, electrodes, and cells. His span from fundamental studies of the understanding of the main phenomena and challenges in the field and through experimental and theoretical studies, all the way to applied work. He holds more than 100 patents and publications and manages numerous company and government research grants*.

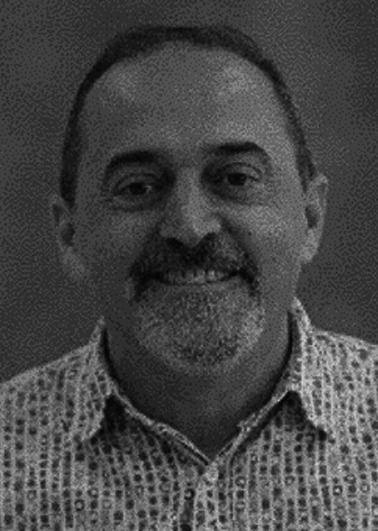

## Biographical Information

*Frédéric Jaouen is since 2013 a Researcher at the Centre National de la Recherche Scientifique, Institut Charles Gerhardt de Montpellier. He obtained his Ph.D. at the Royal Institute of Technology of Stockholm in 2003, on the modeling and experimental characterization of Pt‐based cathodes. He was then a research associate in the group of Prof. Dodelet (Varennes, Canada) from 2004 to 2011, focusing on non‐noble metal catalysts for the oxygen electro‐reduction. His work focuses on platinum‐group metal‐free electrocatalysts, from their synthesis to their characterization ex situ and operando, and their application for key electrochemical reactions such as the O_2_ electro‐reduction, CO_2_ electro‐reduction, and oxygen evolution reaction*.